# Magnetic Energy Losses and Temperature Control System for Giant Magnetostrictive Transducer

**DOI:** 10.3390/mi14010177

**Published:** 2023-01-10

**Authors:** Yafang Li, Xia Dong, Xiaodong Yu

**Affiliations:** School of Information and Automation Engineering, Qilu University of Technology (Shandong Academy of Sciences), Jinan 250353, China

**Keywords:** giant magnetostrictive transducer, high-frequency, energy loss, temperature

## Abstract

The giant magnetostrictive transducer (GMT) can be widely used in ultra-precision machining in precision-fluid-control fields. The temperature stability of GMT is critical for the reliable generation of output characteristics. This study presents a magnetic-energy-losses method for the GMT working at high frequency, and designs a temperature-stable control system to improve energy transmission and heat dissipation. Based on the loss-separation theory and experimental data, the temperature-rise characteristics of the transducer are analyzed. The temperature rise considers the effects of hysteresis loss, the eddy-current loss, the anomalous loss and the Joule heat. A constitutive relation among losses, frequency and magnetic-flux density is given. The temperature distribution of the transducer can be quickly and accurately calculated, using the constitutive equation. According to the convective heat-transfer and the thermal-compensation method, a temperature-control system is designed. A prototype of the system is then fabricated and tested to verify the feasibility and efficacy of the proposed design methods. The results demonstrate that the output- displacement deviation can be controlled at less than 0.65 μm, and the temperature difference is less than 3 °C.

## 1. Introduction

Terfenol-D (Tb_0.27_Dy_0.73_Fe_2_) is a kind of magnetostrictive material, which has large magnetostriction (2000 ppm), giant energy density (25 kJ/m^3^) and high thermal conductivity (13.5 w/(m·k) at 20 °C). It is the core component of the giant magnetostrictive transducer (GMT), which can be widely used in ultra-precision machining in precision-fluid- control fields [1,2]. The performance of the GMT is determined by the precision of the GMT output-displacement. When the GMT is excited by a high-frequency magnetic field, the output displacement consists of magnetostrictive strain and thermal-expansion strain caused by temperature change. The temperature of the Terfenol-D rod can reach above 120 °C without a cooling device. Due to the temperature sensitivity of Terfenol-D, it needs to work under a certain range of temperature to improve the magnetostrictive strain and optimize the device output-displacement. For the design and controlling of high-frequency GMT, the accurate prediction of magnetic energy losses is essential. Therefore, research into the mechanism of temperature rise and determining how to restrain and control the thermal-deformation displacement are difficult points and key technologies.

To predict the dynamic-loss behavior of ferromagnetic material, on the basis of the finite element method, Fallah et al., presented an approach for the hysteresis using the finite element analysis of the 2-D diffusion equation [3]. Do et al., proposed a 3-D finite element multi-physics model, which included a multi-scale model of non-linear magnetostrictive materials for the magnetostatic biasing regime and took into account the effect of the eddy currents for the dynamic regime [4]. On the basis of the free-energy method, Evans et al., presented an energy-weighted averaging class of magneto-mechanical models by developing an efficient implementation for magnetic hysteresis [5]. Xu et al. presented a magneto-elastic coupling dynamic-loss hysteresis model for giant magnetostrictive materials, considering the eddy-current loss and anomalous loss [6]. In addition, Talebian et al. studied classical and excess eddy-currents losses of Terfenol-D and the effects of magnetic field frequency [7]. In references [3,4,5,6,7] established loss models to predict the loss characteristics of materials. For the magnetic properties of magnetostrictive materials, Huang et al. focused on temperature as a factor to study the variation of magnetic properties and loss characteristics of various magnetostrictive materials [8]. Birčáková et al., presented a wide frequency and magnetic-field-range dependencies of core losses and permeability in magnetostrictive materials [9]. For the study of the temperature-rise mechanism, previous works mainly concentrate on dynamic losses of materials [3,4,5,6,7,8,9]. The relationship among the losses of the transducer, frequency and magnetic field is not analyzed.

To establish nonlinear constitutive models for magnetostrictive materials, Zheng et al., proposed a nonlinear constitutive model (Zheng Xiao-Jing model). The model can effectively capture the mechanical-magnetic coupling characteristics of the magnetic, magnetostrictive and elastic properties of Terfenol-D rods. Furthermore, this research group proposed an electromagnetic-mechanical-thermal coupling model for magnetostrictive materials [10,11]. Based on the Jiles–Atherton model and the Zheng Xiao-Jing model, Hu et al. presented a nonlinear magnetomechanical-coupling constitutive model. The model introduces the global coupling-factor related to the magnetomechanical coupling into magnetostrictive strain [12].

For the research on the temperature-control scheme to reduce the temperature rise caused by the dynamic losses, Kwak et al., presented a temperature-control device using air to cool transducers [13]. Zhu et al., developed a precise heat-induced displacement-suppression system which consists of a temperature-control module and a thermal-displacement-compensation module [14], but the heat source of the high-frequency GMT is not accurately calculated. Cai et al., presented an equivalent-circuit model for a transducer, in which the total-electrical-impedance equation is obtained, but the cooling system of the transducer is not made [15]. Zhou et al., proposed a cooling method of spiral-tube winding, and verified its cooling performance via vibration experiments [16]. Ma et al., developed a separated rotary giant-magnetostrictive-ultrasonic system, with a simpler mechanical structure, an energy-transmission structure, and a heat-dissipation structure [17]. However, this system is not suitable for high-frequency and high-power transducers.

To solve the above problems, based on the loss-separation theory, a magnetic-energy-loss model of Terfenol-D at high frequency is proposed. The hysteresis loss coefficients, the eddy-current loss coefficient, and the anomalous loss coefficient can be calculated. The heat source of the high frequency GMT is accurately determined by the model. A temperature-control system is designed and applied to a high-frequency GMT (*f* > 6000 Hz). The accuracy and feasibility of the proposed approach are tested by the calculated results and the experimental ones.

## 2. Magnetic-Energy Losses of Giant Magnetostrictive Transducer at High Frequency

### 2.1. Finite Element Model for Giant Magnetostrictive Transducer

According to Fourier’s heat-transfer theory, the governing equation of the thermal field can be mathematically modeled as follows:

(1) $$\int_{V}\rho C_{p}\cdot \frac{\partial T}{\partial t}\cdot \delta TdV+\int_{V}k\cdot \nabla T\cdot \nabla \delta TdV=\int_{\partial V}k\nabla Tn\cdot \delta Td\partial V+\int_{V}Q\cdot \delta TdV$$

where *T* is the temperature, *k* is the thermal conductivity, *ρ* is the density, *C_p_* is the specific heat capacity, *V* is the volume, *n* is the normal vector of the boundary surface, *Q* is the heat source, *∂V* is the boundary enclosing the volume. The weighted quantity, δT, is multiplied and integrated into the whole domain. Based on the governing equation, a simulation of the whole GMT working process is programmed, using finite element software. In Equation (1), the heat source *Q* includes the losses of the Terfenol-D rod and the Joule heating of the driving coils *p_J_*. The losses of the Terfenol-D rod include the effects of hysteresis loss, *p_h_*, the eddy-current loss, *p_e_*, and the anomalous loss, *p_a_*.

(2) $$Q=p_{h}+p_{e}+p_{a}+p_{J}$$

The hysteresis loss, the eddy-current loss and the anomalous loss are determined by the loss-separation theory [18,19,20]

(3) $$p_{h}=k_{h}fB_{m}{}^{\partial }$$

(4) $$p_{e}=k_{e}f^{2}B_{m}{}^{2}$$

(5) $$p_{a}=k_{a}f^{\frac{3}{2}}B_{m}{}^{\frac{3}{2}}$$

where *k_h_* and ∂ are the hysteresis loss coefficients, *k_e_* is the eddy-current loss coefficient, *k_a_* is the anomalous loss coefficient, *f* is the frequency of the driving magnetic field, and *B_m_* is the maximum magnetic-flux density. For high-frequency applications of Terfenol-D, these coefficients are not known exactly. They need to be identified by experiments.

The Joule heating of the driving coils can be expressed by

(6) $$p_{J}={\left(I_{b}+\sqrt{2}I_{d}sin2\pi ft\right)}^{2}R$$

where *I_b_* is the bias current, *I_d_* is the root-mean-square value of the driving current, and *R* is the resistance of the coil.

### 2.2. Magnetic-Energy Losses of Terfenol-D

**Figure 1:**
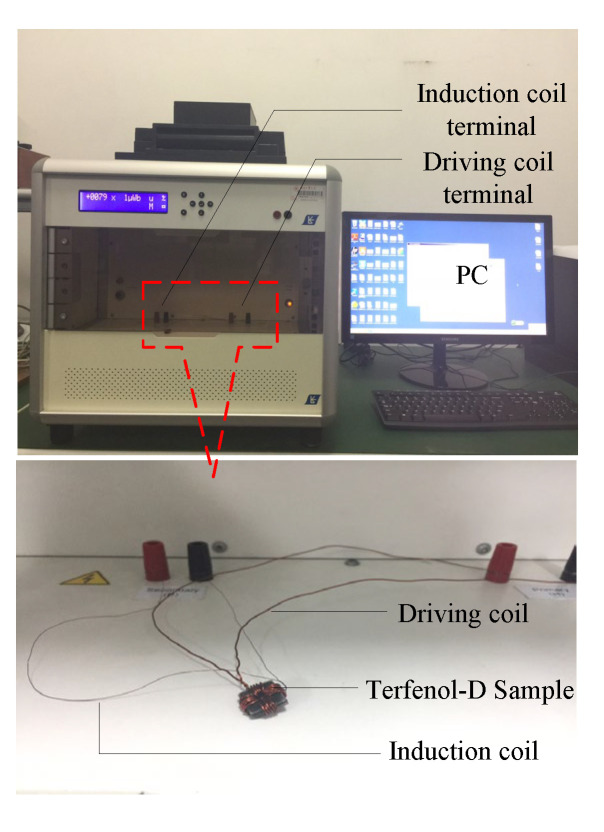
Dynamic-hysteresis-loop measurement system.

Of crucial importance for the transducer design is to quickly and accurately analyze and calculate the energy losses. Based on the loss-separation theory and experimental data of dynamic hysteresis, a constitutive relation among losses, frequency and magnetic flux density can be given. By building a dynamic-magnetic-characteristic test platform, the dynamic-magnetic characteristics of magnetostrictive material are tested and analyzed. The dynamic-hysteresis measurement system (AMH-1 M-S) is shown in Figure 1. It can measure the dynamic-hysteresis curve of magnetic materials. The Terfenol-D sample is the Tb_0.27_Dy_0.73_Fe_2_ alloy. The dimensions of the Terfenol-D specimen are an external diameter of 10 mm, internal diameter of 6 mm and height of 2 mm. The turns of the driving coil and induction coil are 20 and 10, respectively.

The signal generator and power amplifier provide an alternating magnetic field for the driving coil. The induced electromotive force is generated in the induction coil. The magnetic field in the Terfenol-D sample is obtained from the voltage on sampling resistance. The integral amplifier circuit is connected to the induction coil, and the magnetic flux density in the Terfenol-D sample is reflected by the capacitive voltage in the amplifier circuit. The oscilloscope collects the driving-coil signals through the voltage on sampling resistance and the induction-coil signals through the capacitive voltage, simultaneously. The maximum magnetic-flux density, the coercivity and the remanence can be obtained from the measured dynamic-hysteresis curves. The magnetic loss can be obtained by calculating the area of the hysteresis loop.

**Figure 2:**
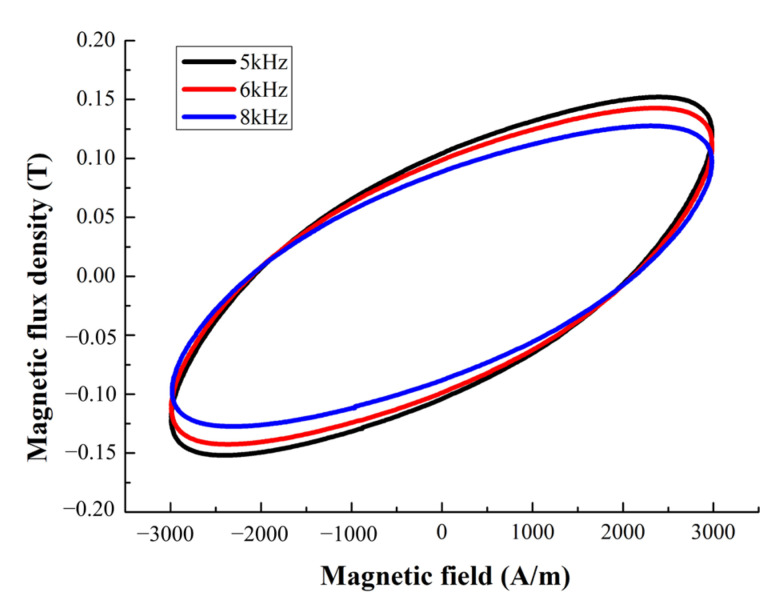
Dynamic-hysteresis curves at different frequencies.

When the maximum magnetic field is 3 kA/m, the dynamic-hysteresis curves at different frequencies (5000 Hz, 6000 Hz, 8000 Hz) are shown in Figure 2. The losses of the hysteresis loop are 363.7 W/kg, 414.9 W/kg, 497.172 W/kg, respectively. It can be seen that when the frequency increases the losses show an increasing trend, but the maximum magnetic-flux density and the remanence gradually decrease.

**Figure 3:**
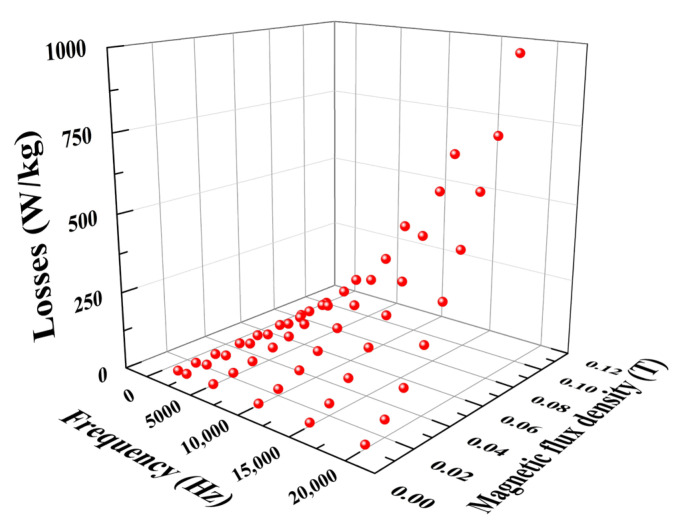
Losses at different frequencies and magnetic-flux densities.

In order to determine the hysteresis loss coefficients *k_h_* and ∂, the eddy-current loss coefficient *k_e_*, and anomalous loss coefficient *k_a_* in Equations (3)–(5), we carried out 54 group experiments of dynamic hysteresis. When the driving frequency was 1000 Hz, 2000 Hz, 5000 Hz, 10,000 Hz, 15,000 Hz, 20,000 Hz respectively, and the maximum magnetic-flux density was 0.01 T, 0.02 T, 0.03 T, 0.04 T, 0.05 T, 0.06 T, 0.07 T, 0.08 T and 0.09 T respectively, the dynamic-hysteresis curves were measured. The losses of the hysteresis loop were obtained, as shown in Table 1 and Figure 3.

Based on physical reasons of the loss-separation model and the data in Figure 3, the parameters *k_h_*,∂, *k_e_* and *k_a_* were fitted through the 1stOpt software. The data fitting was repeated 10 times. The average fitting coefficients were calculated. The losses of the Terfenol-D rod per unit mass were

(7) $$Q_{loss}=p_{h}+p_{e}+p_{a}=2.558fB_{m}{}^{1.9}+1\times 10^{-4}f^{2}B_{m}{}^{2}+1.9\times 10^{-3}f^{\frac{3}{2}}B_{m}{}^{\frac{3}{2}}$$

**Figure 4:**
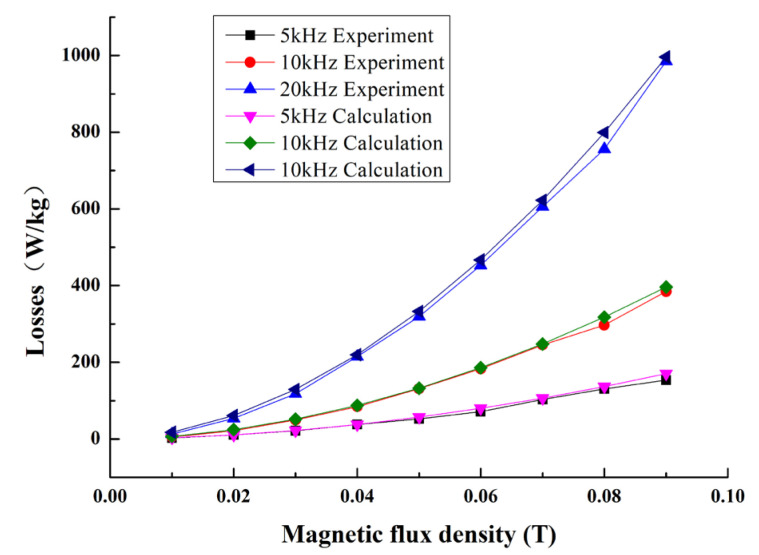
Experiment and calculation of energy losses (5 kHz, 10 kHz and 20 kHz).

Equation (7) is a constitutive relation among losses, frequency and magnetic-flux density. Using Equation (7), the magnetic-energy losses of Terfenol-D can be calculated. Figure 4 shows the calculated and experimental losses of Terfenol-D at different frequencies and magnetic-flux density. It can be seen that the experimental result is in good agreement with the calculated one, indicating that the proposed model accurately describes the losses of the Terfenol-D material. At the same time, the constitutive equation can provide theoretical support for the temperature analysis of the magnetostrictive transducer.

**Figure 5:**
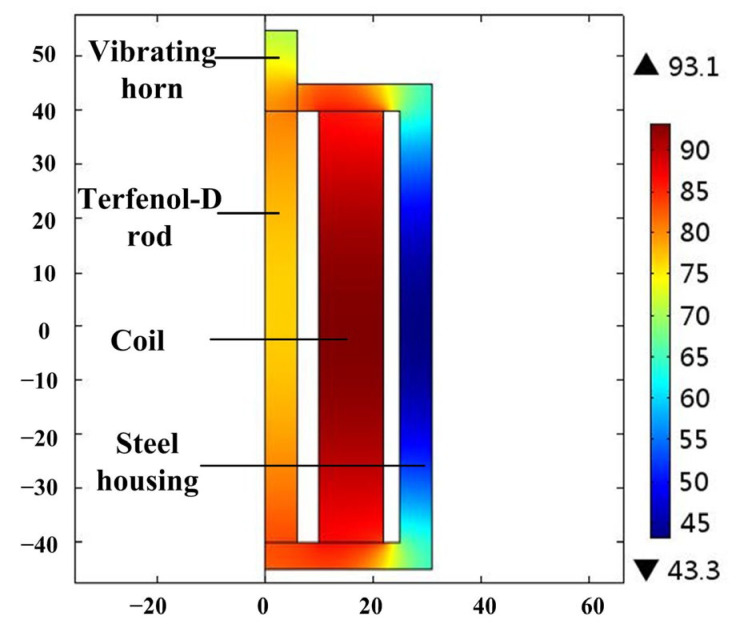
Temperature distribution of the GMT.

According to Equations (1)–(7), the heat source, *Q*, of the GMT at different frequencies and magnetic-flux densities can be accurately calculated. The temperature distribution of the GMT is calculated at 8A bias current, 2A driving current and 6000 Hz, as shown in Figure 5. At these conditions, the losses of the Terfenol-D rod are 97.9 W, and the Joule heat loss of the driving coils is 34.7 W. The result shows that the temperature of the Terfenol-D rod exceeds 80 °C when the GMT works for 30 min, and the temperature of the coil exceeds 93 °C. The temperature rise seriously affects the GMT performance.

## 3. The Prototype of Giant Magnetostrictive Transducer and Testing System

### 3.1. The Structure and Materials of Giant Magnetostrictive Transducer

**Figure 6:**
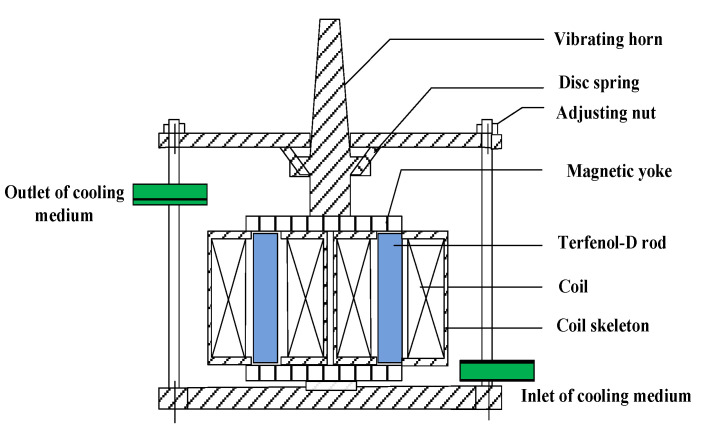
The prototype of the GMT.

A GMT is designed and fabricated with an internal double-cavum cooling channel. The 2-D section of the main part of the GMT is shown in Figure 6. The GMT consists of two Terfenol-D rods (Tb_0.27_Dy_0.73_Fe_2_), driving coils, a disk spring, an adjusting nut, a vibrating horn, a magnetic yoke, and a shell. The structure parameters of the GMT are presented in Table 2.

The Terfenol-D rod produces axial deformation under a driving magnetic field. It converts electromagnetic energy into mechanical energy. The Terfenol-D rod is surrounded by driving coils and bias coils. The magnetic circuit is closed through a magnetic yoke. The disc spring and the adjusting nut provide prestress for the Terfenol-D rod. A temperature control system of the GMT is also shown in Figure 6. According to the convection-heat-transfer theory and the thermal-compensation method [21], in the GMT there is an internal double-cavum cooling channel. The cooling channel is formed between the coil skeleton and the Terfenol-D rod, the coil skeleton and the external steel housing, where the temperature rise of the GMT is the most serious. The cooling medium is silicone oil, which is refrigerated by a circulating cooler. The inlet of the silicone oil is designed to be at the bottom, and the outlet at the top. This design can reduce the increase in temperature of the transducer more effectively. The Terfenol-D used in the temperature-control system is directly exposed to silicone oil, thereby ensuring a low working temperature and an optimal environment.

### 3.2. The Temperature-Control Testing System

**Figure 7:**
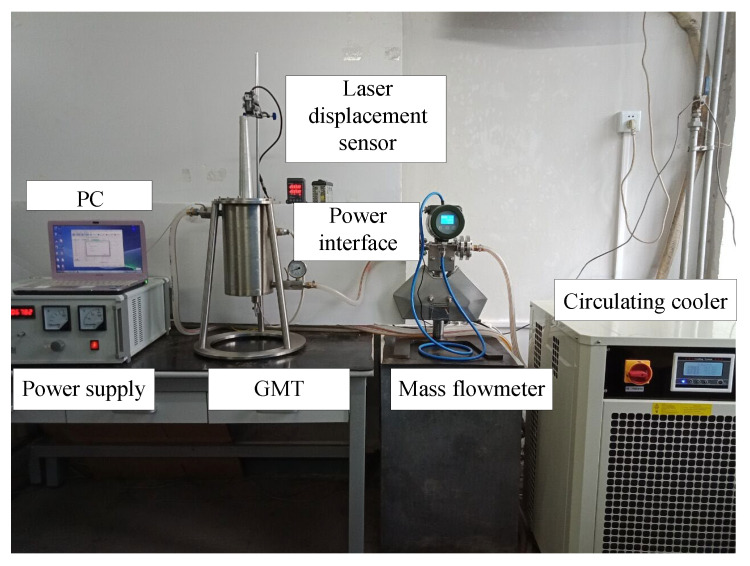
Temperature-control system of the GMT.

The GMT prototype and the temperature-control testing system are shown in Figure 7. The temperature-control device includes the internal double-cavum cooling channel, the mass flowmeter (YK-LK-10-FT51S, Yoke Instrument, Dalian, China) and the circulating cooler (Shanghai TOYO, Shanghai, China). A PC and the temperature-control device form a closed-loop control system. There are temperature sensors at the inlet and outlet, which can record real-time temperature. The flow velocity of the silicone oil can be controlled by adjusting the circulating cooler. The mass flowmeter is used for monitoring the velocity of the silicone oil. The output displacement of the GMT can be measured using a laser displacement sensor (KEYENCE, Beijing, China).

To verify the efficacy of the design of the proposed temperature-control system in terms of the performance improvement of its temperature stability, an experimental evaluation was performed on a prototype of the designed GMT.

## 4. Results

**Figure 8:**
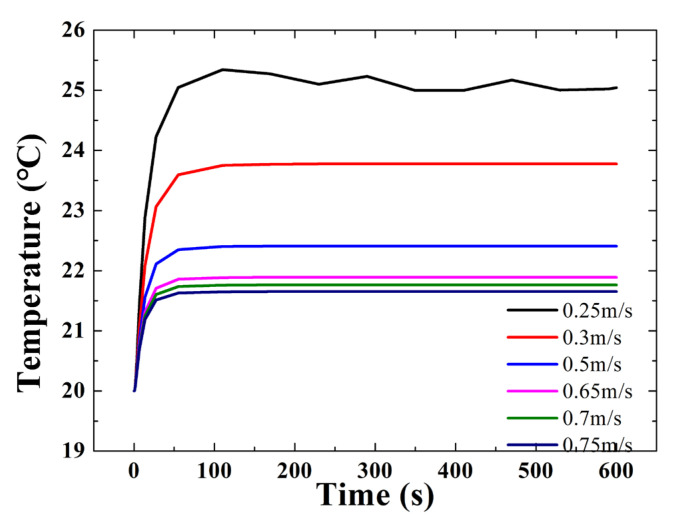
Temperatures of the Terfenol-D rod vs. the flow velocity of silicone oil.

The relationship between the inner temperature of the GMT and the flow velocity of the silicone oil is analyzed. The temperature of the Terfenol-D rod is mainly considered. The temperature of the Terfenol-D rod vs. the flow velocity of the silicone oil is shown in Figure 8. When the flow velocity of the silicone oil *v* = 0.25 m/s, the temperature can be controlled at 25 °C. The temperature decreases with the increase in flow velocity. When *v* = 0.5 m/s, the temperature can be controlled to within 22.3 °C. It can be seen that, with the increase in flow velocity, the temperature decreases slowly when *v* > 0.5 m/s. At the same time, the pressure of the internal cavum of the transducer needs to be considered. The pressure should be less than 0.1 MPa. Above all, the flow velocity of the silicone oil *v* = 0.5 m/s is selected as the most suitable flow velocity.

**Figure 9:**
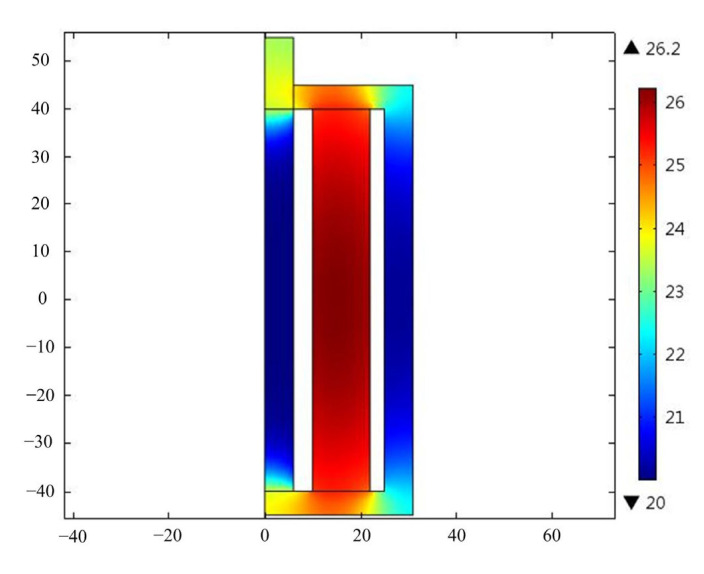
Temperature distribution of the GMT with temperature control.

Figure 9 shows the temperature distribution when the GMT uses the designed temperature-control system. The working conditions are also 8A bias current, 2A exciting current and 6000 Hz frequency. The initial cooling temperature is set at 20 °C. The results show that the temperature of the coil can be controlled below 26.2 °C. The temperature at the top of the Terfenol-D rod is controlled at 22.4 °C. Specifically, the temperature at the center of the Terfenol-D rod is lower, at 21.1 °C.

**Figure 10:**
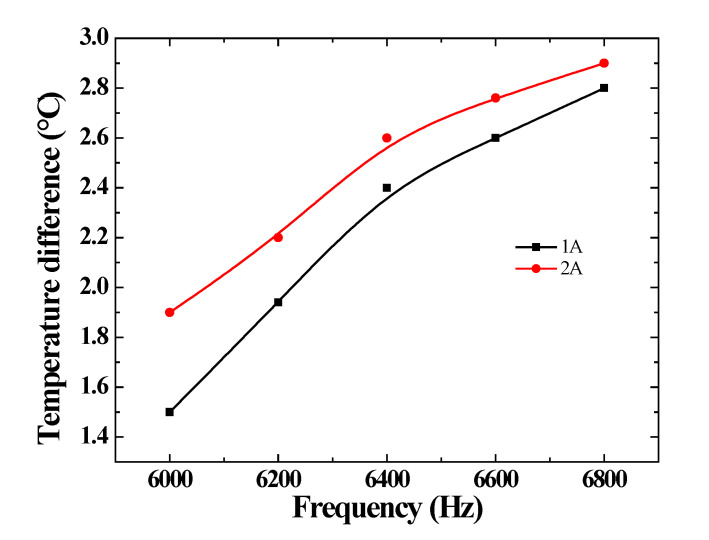
Temperature difference between the outlet and the inlet.

To verify that the temperature-control system can work well at high frequency, by collecting data from the temperature sensors the temperature differences between the outlet and the inlet are analyzed. The experimental conditions are as follows: the driving current is 1A and 2A, the bias current is 8A, the cooling temperature of the inlet is 20 °C, and the flow velocity of the silicone oil is 0.5 m/s. The frequency is 6000 Hz, 6200 Hz, 6400 Hz, 6600 Hz and 6800 Hz, respectively. The experimental results are exhibited in Figure 10. When the frequency and driving-current increase, the temperature differences become larger. The temperature difference is 1.5 °C at 6000 Hz and 1A driving current. It increases to 2.9 °C at 6800 Hz and 2A driving current. The results indicate that the temperature difference is less than 3 °C, using the designed system. Therefore, this temperature-control system can provide a suitable temperature environment for the GMT.

**Figure 11:**
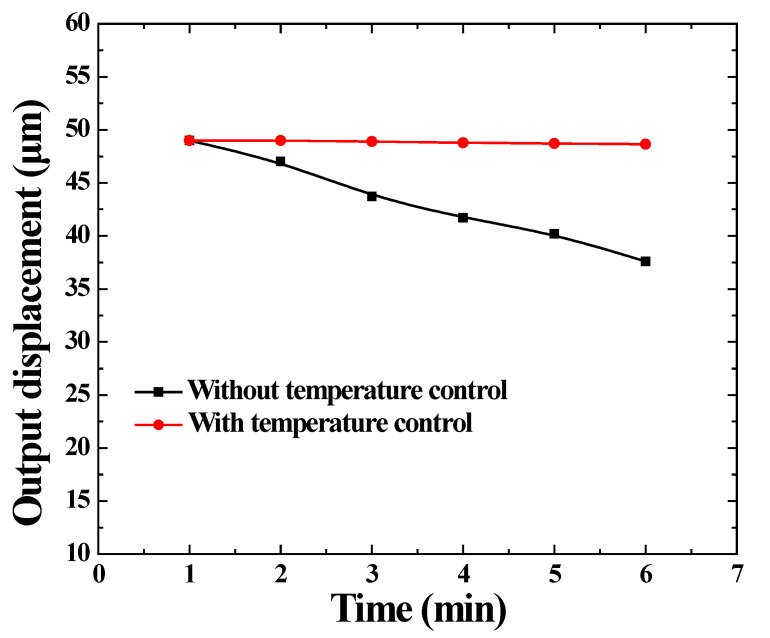
Output displacement of the GMT.

The output displacement consists of two parts: (a) Magnetostrictive strain. The magnetostriction decreases when the temperature increases by 1000 ppm at 20 °C, and drops to 890 ppm at 40 °C. For this reason, as the temperature increases, the output displacement decreases. (b) Thermal expansion strain. This is caused by temperature change, and can be calculated by $y_{T}=\alpha_{T}\Delta T$, where $\alpha_{T}$ is the thermal expansion coefficient. The thermal expansion strain increases the output displacement. In fact, the influence of temperature on the magnetostrictive strain is larger than that of the thermal expansion strain. Therefore, the output displacement decreases as the temperature rises. The amplitudes of the output displacement of the GMT are measured by the laser displacement sensor, as shown in Figure 11.

The amplitudes of the output displacement are measured at a working of 1–6 min when the GMT with the temperature-control system works at 6000 Hz, a 2A driving current and 0.5 m/s flow velocity of the silicone oil. The maximum amplitude is 49 μm at continuous working of 1 min. The minimum amplitude is 48.35 μm at a continuous working of 6 min. It can be concluded that the output-displacement deviation is controlled for less than 0.65 μm. The deviation is 1.3%. When there is no temperature-control system, it can be found that the output displacement has a significant decline, from 49 μm to 37.6 μm (declined by 23.3%). The output accuracy of the GMT is obviously affected. These data indicate that this temperature-control system is reasonable designed, and it is effective for the GMT working at high frequencies.

## 5. Conclusions

A temperature-control approach is proposed in this paper, to increase the accuracy of the performance of the GMT. In order to improve the prediction accuracy of the magnetic energy losses, we first use the loss-separation theory to determine the high-frequency hysteresis loss coefficients, the eddy-current loss coefficient, and the anomalous loss coefficient. Then, we determine the explicit formula for the relationship among losses, frequency and magnetic-flux density, and finally we use Fourier’s heat-transfer theory to accurately calculate the inner temperature rise of the high-frequency GMT. Based on the convection-heat-transfer theory and the thermal-compensation method, a temperature-control system is designed. The accuracy and feasibility of the proposed approach are tested by the calculated results and the experimental ones. The temperature of the GMT is controlled to below 26.2 °C, and the output-displacement deviation can be controlled at less than 0.65 μm (1.3%) when the frequency is 6000 Hz. These results can be used in the optimal design, estimation and control of the GMT system at a higher frequency.

## Figures and Tables

**Table 1:** Losses at different frequencies and magnetic-flux densities.

Losses (W/kg)	1000 Hz	2000 Hz	5000 Hz	10,000 Hz	15,000 Hz	20,000 Hz
0.01 T	0.466	1.048	2.742	5.392	10.728	12.671
0.02 T	1.573	3.66	10.844	21.581	38.413	53.692
0.03 T	3.206	7.438	21.464	49.596	86.843	117.612
0.04 T	5.774	13.543	37.99	84.357	151.549	214.641
0.05 T	8.138	19.482	52.248	131.309	227.723	319.482
0.06 T	11.948	27.245	71.688	183.076	313.087	453.147
0.07 T	16.112	32.574	103.005	245.504	432.511	605.87
0.08 T	17.862	43.584	130.33	297.08	555.802	756.442
0.09 T	23.375	45.571	153.89	384.58	659.922	985.017

**Table 2:** Main Materials and Parameters of the GMT.

Parameter	Value	Parameter	Value
Terfenol-D rod	2 rods	Coil turns	150 turns
Terfenol-D rod radius	7.5 mm	Coil bobbin length	120 mm
Terfenol-D rod length	102 mm	Coil outward radius	14 mm
Horn-top diameter	28 mm	Horn length	310 mm
Horn-bottom diameter	67 mm	Shell radius	76 mm
Magnetic-yoke length	60 mm	Magnetic yoke width	16 mm

## Data Availability

Not applicable.
